# An annotated haplotype-resolved genome sequence assembly of diploid German chamomile, *Matricaria chamomilla*

**DOI:** 10.1038/s41597-025-04688-4

**Published:** 2025-02-28

**Authors:** Woohyeon Cho, Jiawu Feng, Manuela Knauft, Sebastian Albrecht, Axel Himmelbach, Lars-Gernot Otto, Martin Mascher

**Affiliations:** 1 Leibniz Institute of Plant Genetics and Crop Plant Research (IPK) Gatersleben, 06466 Seeland, Germany; 2 Department of Agriculture, Forestry and Bioresources, Plant Genomics and Breeding Institute, College of Agriculture and Life Sciences, Seoul National University, 1 Gwanak-ro, Gwanak-gu, Seoul, 08826 South Korea; 3 PHARMAPLANT GmbH, Arznei- und Gewürzpflanzen Forschungs- und Saatzucht GmbH, Am Westbahnhof 4, D-06556 Artern, Germany

**Keywords:** Plant genetics, Plant breeding

## Abstract

*Matricaria chamomilla* L. (chamomile) is a medicinal plant that is widely used for treating skin infections and respiratory ailments. Chamomile belongs to the Asteraceae family of flowering plants and is a primarily outcrossing species with a heterozygous genome. Despite its extensive use, no reference genome has been available for chamomile until now. We present a chromosome-level genome sequence for chamomile which was assembled with TRITEX pipeline from PacBio accurate long reads and chromosome conformation capture sequencing data. The assembled pseudo-haploid genome has a total size of 2.75 Gb, organized into 9 chromosomes with a scaffold N50 of 285 Mb. This high-quality reference genome has a BUSCO value of 98.8% and includes 47,820 functional genes. Additionally, we assembled a haplotype-resolved genome, taking advantage of the high heterozygosity of chamomile. The haplotype assemblies have total sizes of 2.28 Gb and 2.34 Gb and cover 87.6% and 89.8% of the pseudo-haploid reference genome, respectively. Our assemblies provide a valuable resource for genetics and genomics works for chamomile and related members of the Asteraceae.

## Background & Summary

*Matricaria chamomilla* L. (syn. *Matricaria recutita* L., True or German chamomile, hereafter chamomile) is one of the most important medicinal plants worldwide, with a wide range of medical applications like infections and inflammatory diseases of the skin, gastrointestinal diseases and respiratory problems, and a long history of use^1^. *Matricaria chamomilla* belongs to the Asteraceae (syn. Compositae), one of the largest plant families consist of approximately 23,600 species. Chamomile is mainly an outcrossing species^2,3^. This is why individual chamomile genomes are heterozygous and chamomile varieties are heterogeneous mixtures of genotypes. To complicate matters furthers, chamomile genotypes can be either diploid or tetraploid^4^, with a chromosome number of 9 for the haploid genome^5^.

Despite rapid progress in the cost-efficiency and quality of the sequencing technologies, high quality whole-genome sequence assembly have been published hitherto for only a few medicinal and aromatic plants from the Asteraceae family such as sunflower (*Helianthus annuus*, approx. 3.6 Gb genome size^6^), *Artemisia annua* (approx. 1.7 Gb genome size^7^), or *Chrysanthemum makinoi* (approx. 3.2 Gb genome size^8^). No genome assembly is available for chamomile, yet.

In this study, we obtained a high-quality pseudo-haploid sequence assembly as well as haplotype-resolved genome sequences of a diploid *Matricaria chamomilla* individual. To do so, we used PacBio HiFi and Hi-C data and assembled the data with the TRITEX pipeline^9^. The generated draft genome assembly provides a very valuable resource for genetics and genomics works at chamomile.

## Methods

### Materials and sequencing

One single diploid chamomile genotype from the variety ‘Bona’ was chosen for the genome assembly. ‘Bona’ is an agriculturally well performing variety, from which an MS-line was generated, which could be very valuable for further chamomile breeding^10^. Clonal plants of this genotype were preserved in *in vitro* culture at 10 °C under short-day conditions (8 h light), until raised for DNA isolation in the greenhouse under long-day conditions (16 h/8 h light/dark period with 22 °C/16 °C). The ploidy for each clone was analysed by flow-cytometry according to Otto *et al*. 2015 and confirmed as diploid^11^. For the isolation of high molecular weight (HMW) DNA, 7 g leaves were collected from three clonal plants, ground into a fine powder using liquid nitrogen, and stored at -80°C. Approximately 350 µg HMW DNA was purified from the powder using a method as described^12^. For quantification, the Qubit dsDNA High Sensitivity assay kit (Thermo Fisher Scientific, MA, USA) was used. The DNA size profile was analyzed using the Femto Pulse system and the Genomic DNA 165 kb kit (Agilent Technologies Inc, CA, USA). For fragmentation of the HMW DNA (10 µg) into 20 kb fragments, a Megaruptor 3 device (speed: 30) was used (Diagenode, NJ, USA). HiFi SMRTbell libraries were prepared according to the manufacturer’s instructions using the SMRTbell Express Template Prep Kit (Pacific Biosciences, CA, USA). The final HiFi libraries were size-selected (narrow size range: 18–21 kb) using the SageELF system with a 0.75% Agarose Gel Cassette (Sage Sciences, MA, USA) according to standard manufacturer’s protocols. HiFi CCS reads were produced operating the PacBio Sequel IIe instrument (Pacific Biosciences, CA, USA) following the manufacturer’s instructions. In total, 7,281,234 reads corresponding to 163 Gb (HiFi CCS) were generated. The *in situ* Hi-C library preparation was based on the previously published protocol^13^. Sequencing and Hi-C raw data processing was performed as described before^14,15^. A total of 3,710,914,879 reads were generated (about 408 Gb).

### Genome size estimation

**Fig. 1:**
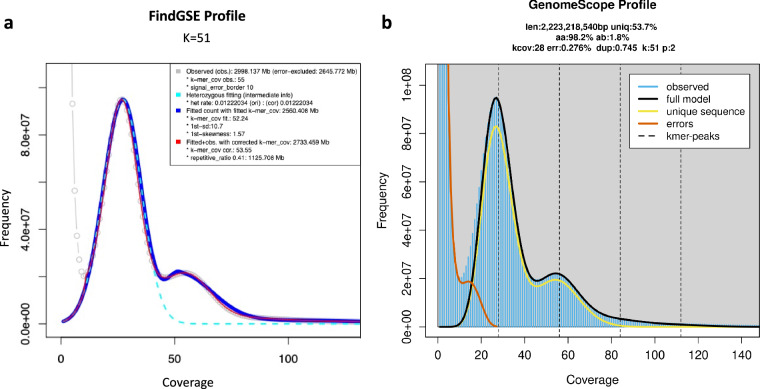
Genome size estimation by K-mer frequency calculation. K-mer profiles were evaluated with FindGSE (**a**) and GenomeScope (**b**).

The genome size was estimated by counting k-mer of HiFi data with word size 51 using Jellyfish^16^. A k-mer frequency histogram was also generated with Jellyfish. Genome size and heterozygosity was estimated using findGSE (https://github.com/schneebergerlab/findGSE). The estimated genome size is 2.73 Gb with heterozygosity rate of 1.2% and repetitive rate of 41% (Fig. 1a). To determine its ploidy, we analyzed the data using GenomeScope^17^ and confirmed it to be diploid (Fig. 1b).

### Pseudo-haploid genome assembly

**Fig. 2:**
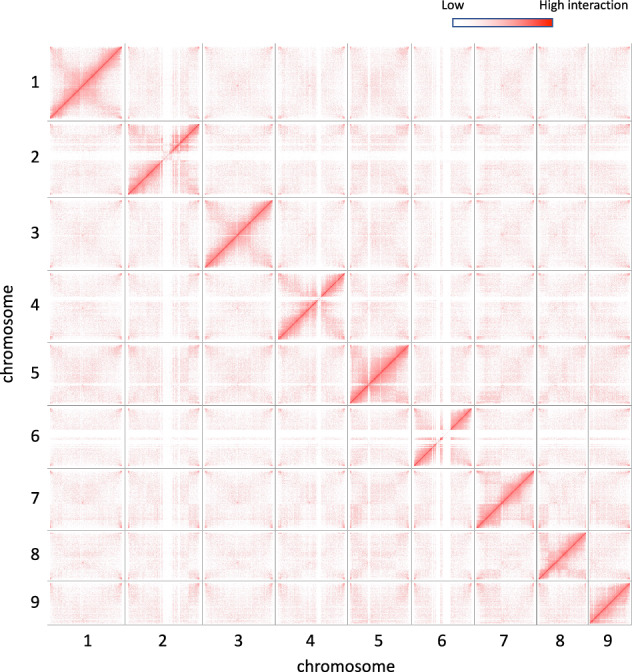
Inter-chromosomal Hi-C heatmap of pseudo-haploid assembly.

The TRITEX long-read assembly pipeline^9^ was used for chromosome-scale pseudo-haploid assembly. Contig assembly was performed using hifiasm with Hi-C and HiFi reads, resulting in 2,662 contigs with an N50 of 50 Mb and a total length of 2.75 Gb (Table 1). To exclude bacterial contamination, we filtered 38 contigs using FCS-GX^18^, leaving 2,624 contigs in the resulting dataset. Hi-C reads were mapped to this contig assembly, with 77,998,277 read pairs mapped. After manual curation as described by Marone *et al*.^9^, the final assembly comprised 2,541 scaffolds, with an N50 of 285 Mb. Out of the 2,624 contigs, 92 contigs were anchored to 9 chromosomes with total length of 2.60 Gb, which is 94.6% of total contig assembly (Table 2, Fig. 2). The nine chromosomes are arranged in order from largest to smallest.

**Table 1:** Pseudo-haploid assembly statistics after primary contig assembly and scaffold assembly.

	After primary contig assembly		After scaffold assembly
	Unitig	Contig	Pseudomolecule + unanchored
No. of assembly	28,889	2,662	9 + 2,532
Total length (bp)	5,769,542,610	2,757,670,726	2,752,017,381
N50 (bp)	2,156,804	50,350,694	285,044,652
N90 (bp)	46,254	8,462,871	195,322,941
Maximum length (bp)	22,543,845	141,852,184	348,051,304

**Table 2:** Chromosome length information of reference and haplotype assembly.

Chromosome	Length (Mb)		
	Pseudo-haploid	Haplotype 1	Haplotype 2
chr1	348.1	374.8	339.7
chr2	340.4	290.4	308.0
chr3	325.8	318.7	316.7
chr4	322.3	318.2	305.2
chr5	285.0	98.9	105.9
chr6	279.4	232.7	277.3
chr7	278.3	258.2	257.7
chr8	227.7	210.4	231.9
chr9	195.3	181.0	197.3
Total	2,602.3	2,283.5	2,340.0

### Haplotype-resolved assembly

**Fig. 3:**
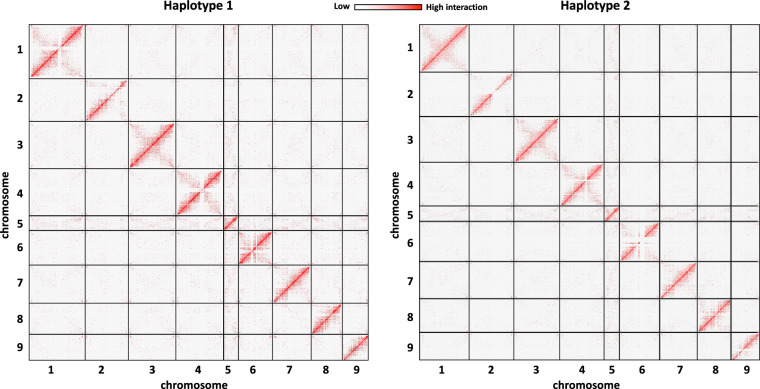
Inter-chromosomal Hi-C heatmap of two haplotypes.

Haplotype-resolved assembly was conducted with the unitig assembly obtained through hifiasm as described by Mascher *et al*.^19^. The unitig assembly contained 28,889 unitigs with an N50 of 2.15 Mb and a total length of 5.76 Gb (Table 1). Total of 4.57 Gb among 5.76 Gb, which is 79% of unitigs were positioned to 9 chromosomes and used for haplotype phasing. Unitigs were phased using PCA with Hi-C linkage information. We found unitigs from homozygous region (i.e. those where both haplotypes are identical in sequence) by read coverage and assigned them to both haplotypes. The final, manually curated pseudomolecules of each haplotype have total lengths of 2.28 Gb (87.6%) and 2.34 Gb (89.8%), respectively (Table 2, Fig. 3). Each chromosome is named according to its corresponding chromosome in the pseudo-haploid genome. We only assembled unitigs larger than 300 Kb, and most of the unitigs assigned to chromosome 5 were smaller than the threshold. As a result, chromosome 5 assembled to 98.9 Mb (34.7% of pseudo-haploid chromosome 5) in haplotype 1 and 105.9 Mb (37.2% of pseudo-haploid chromosome 5) in haplotype 2.

### Pseudo-haploid assembly gene annotation

The EDTA v2.0.0 program was used to annotate the repetitive DNA in the pseudo-haploid sequence assembly^20^. Of a total of 2.75 Gb of sequence, 73.17%, corresponding to 2.01 Gb, were identified as repetitive sequence. The 72.49% of 2.75 Gb which were long terminal repeat (LTR) elements consisted of 27.18% *Copia* elements and 21.82% *Gypsy* elements (Table 3).

**Table 3:** Repetitive elements annotation in pseudo-haploid assembly.

Class		Number of elements	Sequence length (bp)	Percentage of genome
LTR				
Copia		566,348	748,049,544	27.18%
Gypsy		498,678	600,515,119	21.82%
unknown		846,479	646,444,484	23.49%
TIR				
CACTA		2,482	3,123,318	0.11%
Mutator		4,129	4,120,180	0.15%
PIF_Harbinger		532	397,841	0.01%
Tc1_Mariner		600	282,726	0.01%
hAT		7,229	4,337,844	0.16%
nonTIR				
helitron		4,344	6,276,603	0.23%
Total		1,930,821	2,013,547,659	73.17%

To annotate protein-coding genes, we used BRAKER2 with RNA-Seq evidence mode^21^. We downloaded published RNA sequencing data from closely related genera from NCBI. RNA-seq reads of *Chrysanthemum lavandulifolium* (SRR14723013) and *Chrysanthemum nankingense* (SRR18155269, SRR18155270) were adapter trimmed using Trim Galore! (https://github.com/FelixKrueger/TrimGalore), aligned to the genome with HISAT2 (v.2.2.1)^22^, and SAM files were converted to BAM using Samtools (v.1.15.1)^23^. Using all the data, BRAKER2 predicted 236,848 genes. Among them, we obtained only the longest isoform with gtftools (0.5-r234)^24^, resulting in 215,439 genes.

We carried out the functional annotation of genes using a homology-based approach with Blast + (v.2.13.0)^25^. Sequences were compared against the reference proteins (*Arabidopsis thaliana* TAIR10, TAIR10_pep_20101214_updated.fasta.gz - https://www.araport.org) using blastp with an e-value threshold of 1e-10. Among the 215,439 transcripts, 47,820 genes were functionally annotated, having significant homology to proteins in the TAIR database (Table 4).

**Table 4:** Gene annotation in pseudo-haploid assembly.

**Number of annotated genes**	47,820
Total length of genes (bp)	80,605,119
Smallest gene length (bp)	150
Largest gene length (bp)	57,108
Average gene length (bp)	1,685
**Number of unannotated genes**	167,619
**Total number of genes**	215,439

## Data Records

PacBio and Hi-C raw reads can be accessed in the NCBI Sequence Read Archive under project accession number PRJEB64706. PacBio reads are available under accession numbers ERR11788247^26^, ERR11788248^27^, ERR11788249^28^, and ERR11788250^29^. Hi-C reads have been deposited under accession numbers ERR11788245^30^ and ERR11788246^31^. The pseudo-haploid genome assembly sequence has been deposited in the NCBI under the accession number PRJNA1055910 and GenBank accession GCA_963920955.1^32^. The haplotype-resolved genome assembly sequences have been deposited under accession numbers PRJNA1044733 and PRJNA1044734, with GenBank accessions GCA_964234995.1^33^ and GCA 964235185.1^34^, respectively. The gene annotation files are available in Figshare (10.6084/m9.figshare.27037327)^35^.

## Technical Validation

The gene space completeness of the genome assembly was estimated at 98.8% (94.0% single-copied genes, 4.8% duplicated genes, 0.3% fragmented, and 0.9% missing genes) as assessed using BUSCO (v.5.4.6)^36^ with the ‘embryophyta_odb10’ database (n = 1614). The completeness for functionally annotated gene set was 94.6% (88.8% single-copied genes, 5.8% duplicated genes, 2.1% fragmented, and 3.3% missing genes).

**Fig. 4:**
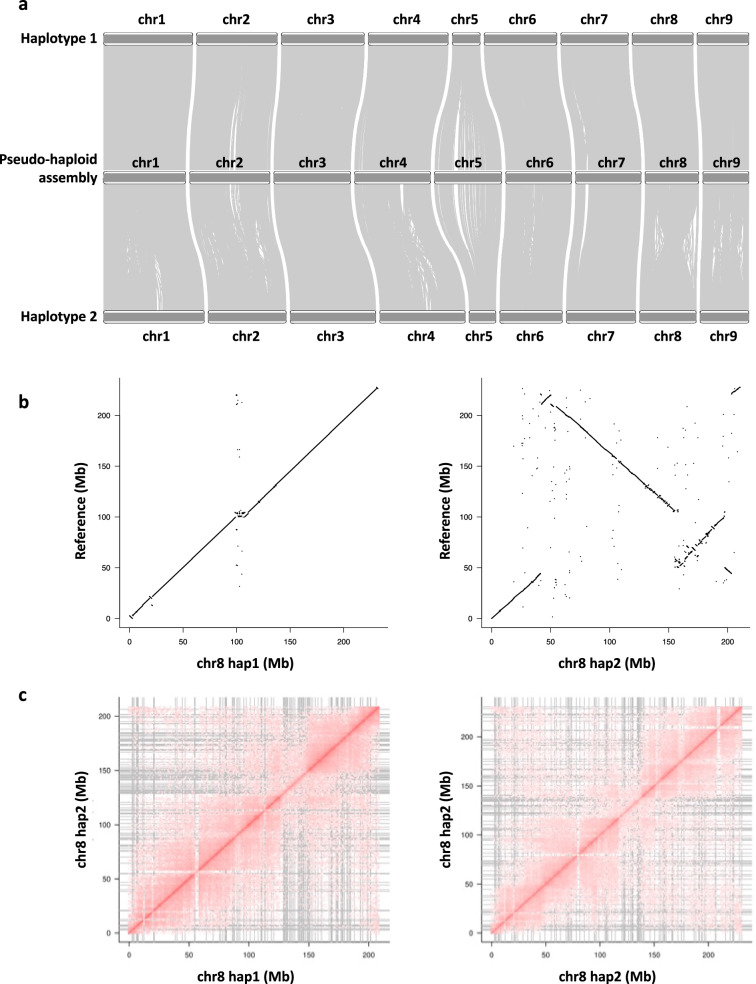
Assembly validation. (**a**) Collinearity between haplotypes and pseudo-haploid genome. (**b**) Collinearity between chromosome 8 of both haplotypes and the pseudo-haploid assembly. (**c**) Hi-C heatmap of chromosome 8 for both haplotypes.

We evaluated the haplotype-phased assembly by plotting the collinearity between pseudo-haploid assembly. Both haplotypes were overall collinear with some structural variants (Fig. 4a). For example on chromosome 8, we observed a large inversion about 150 Mb in size with smaller inversion (~50 Mb in size) nested therein (Fig. 4b). We confirmed inversions by inspecting the Hi-C contact matrices (Fig. 4c), which were devoid of the strong off-diagonal signals characteristic of misassemblies.

## Data Availability

Programs and options used for data processing are described in the ‘Method’ section. For pseudo-haploid genome assembly, we followed long-read TRITEX pipeline^9^. For haplotype-phased assembly, we followed the open-source pipeline (https://github.com/jia-wu-feng/Pan_Bulbosum)^19^. Assembly code modified for this study is available in https://github.com/whyeonc/chamomile-genome-assembly.
